# Gliogenic Potential of Single Pallial Radial Glial Cells in Lower Cortical Layers

**DOI:** 10.3390/cells10113237

**Published:** 2021-11-19

**Authors:** Ana Cristina Ojalvo-Sanz, Laura López-Mascaraque

**Affiliations:** Cellular, Molecular and Developmental Neurobiology Department, Instituto Cajal-CSIC, 8002 Madrid, Spain; anacris23@cajal.csic.es

**Keywords:** *StarTrack*, glial cell types, lineage, progenitor, astrocytes, oligodendroglia, NG2-glia, clonal analysis, cortex, mice

## Abstract

During embryonic development, progenitor cells are progressively restricted in their potential to generate different neural cells. A specific progenitor cell type, the radial glial cells, divides symmetrically and then asymmetrically to produce neurons, astrocytes, oligodendrocytes, and NG2-glia in the cerebral cortex. However, the potential of individual progenitors to form glial lineages remains poorly understood. To further investigate the cell progeny of single pallial GFAP-expressing progenitors, we used the in vivo genetic lineage-tracing method, the *UbC-(GFAP-PB)-StarTrack*. After targeting those progenitors in embryonic mice brains, we tracked their adult glial progeny in lower cortical layers. Clonal analyses revealed the presence of clones containing sibling cells of either a glial cell type (uniform clones) or two different glial cell types (mixed clones). Further, the clonal size and rostro-caudal cell dispersion of sibling cells differed depending on the cell type. We concluded that pallial E14 neural progenitors are a heterogeneous cell population with respect to which glial cell type they produce, as well as the clonal size of their cell progeny.

## 1. Introduction

The development of the cerebral cortex from a pool of Neural Progenitor Cells (NPCs) is an immensely complex process. After the closure of the most rostral part of the neural tube, Neuroepithelial Cells (NECs) undergo symmetric divisions to expand the stem cell pool. Subsequently, NECs lose some epithelial features, differentiating into Radial Glial Cells (RGCs) [1,2,3]. Those RGCs divide symmetrically and/or asymmetrically to sequentially generate pyramidal neurons [4] and then glial cells, including astrocytes, oligodendrocytes, and NG2-glia [5,6,7,8,9,10,11,12]. During brain ontogeny, RGCs are the first cell population that shows glial features, expressing markers such as Glial Fibrillary Acidic Protein (GFAP), Glutamate Aspartate Transporter (GLAST), and Brain Lipid Binding Protein (BLBP) [5]. *GFAP* transcripts can be first detected in mouse brain as early as E9.5–E11 [13,14], with the *GFAP* gene being a candidate to target RGC progenitors [13,14].

Two alternative models could explain the progenitor cell potential [4,6,15]. On the one hand, the “common progenitor” model proposes the existence of a unique progenitor to generate different cell types changing their cell potential over time [16,17,18]. On the other hand, the “multiple progenitor” model suggests the existence of several progenitors committed to a neural cell type [17,18]. Some authors reported glial lineage-restricted progenitors as early as E9.5 [19], while others showed RGC transition to gliogenesis after neurogenesis [20]. In vitro or in vivo cell fate analyses revealed that, at early embryonic stages, only 10–20% of NPCs are bipotent to generate neurons and glia [12,17]. However, transplantation experiments revealed the existence of multipotent progenitors at early stages and progressive neuronal fate restriction along development [21,22]. Therefore, RGCs progressively modify their capacity to proliferate or produce various neural cell types [23] depending on the temporal and regional progenitor diversity [17,19,24,25,26,27]. Although the progression of their molecular identity is determined by both cell-intrinsic and -extrinsic processes [20,28], each progenitor stage displays domain-specific transcription factors [24].

Insights into progenitor diversity have been obtained using genetic lineage-tracking tools, designed to specifically target single NPCs in vivo [18]. At early stages, most RGCs generate neurons in both lower and upper layers [20,29], while at later embryonic stages, one out of six RGCs continues dividing to generate glia [20]. The *StarTrack* method revealed highly specific clonal arrangements for astrocytes (protoplasmic, fibrous, juxtavascular, or pial) [30] and NG2-glia [31] generated from single pallial progenitors at E14. However, *StarTrack* labeling is only visible in the GFAP+ progeny of NPCs, since the fluorescent label is not visible to all the possible cell fates when the GFAP promoter is switched off [30]. Thus, considering that our aim is to analyze the complete cell progeny from GFAP+ progenitors (RGC-progenitors), we used the *UbC-(GFAP-PB)-StarTrack* [32]. This tool is based on the combination of *UbC-StarTrack* plasmids [33] with a hyperactive *PiggyBac* transposase expressed under the *GFAP* promoter. This allows for tracking the complete cell progeny of single RGC progenitors, independently of their lineage [32,33]. Although the neuronal fate of those progenitors is well established [2,4,6,12,22,23,29,34,35], there is little evidence with respect to their glial fates [32,33]. Thus, in this work, we analyzed the glial progeny originating from single pallial E14-RGC progenitors. In addition, due to the existence of transcriptional differences in glial cell types located in lower or upper cortical layers [36,37,38], we focused on glial clones located in lower cortical layers. We demonstrate the existence of adult glial clones containing sibling cells of either one glial cell type (uniform clones) or from two different glial cell types (mixed clones), although many of them just produce a single glial subtype.

## 2. Materials and Methods

### 2.1. Mouse Line

Adult and pregnant wild-type C57BL/6 mice were housed at the animal facility of the Cajal Institute. Mice were handled and treated according to the guidelines of the European Union on the use and welfare of experimental animals (2010/63/EU) and those of the Spanish Ministry of Science and Innovation (RD 1201/2005 and L 32/2007). All the experiments were approved by the Cajal Institute, CSIC, and the Community of Madrid Bioethical Committees (PROEX 314/19). Considering that the mice’s gestation period lasted 19 days, the day of visualization of the vaginal plug was considered as embryonic day (E0) and the day of birth as postnatal day (P0). Adult mice were considered from P30 onwards. *N* = 3 animals were used for the experiments.

### 2.2. DNA Vectors

We used the *UbC-(GFAP-PB)-StarTrack* [32], a novel strategy designed based on the *UbC-StarTrack* [33]. This approach enables us to specifically target GFAP+ progenitors (RGC progenitors) and to follow their complete cell progeny, independently of their lineage. Briefly, the CMV promoter of the hyperactive transposase of the PiggyBac system (CMV-hyPBase) was exchanged for the human promoter of the glial fibrillary acidic protein (GFAP-hyPBase), provided by Dr. Lundberg. To confirm successful cloning, all the plasmids were sequenced (Sigma–Aldrich; Merck KGaA, Darmstadt, Germany). An equal mixture of 12 *UbC-StarTrack* plasmids, GFAP-hyPBase, and *Cre*-ERT2 was co-electroporated in all the experiments.

### 2.3. In Utero Electroporation (IUE) and Tamoxifen Administration

IUEs were performed at E14 [30,33] in pregnant mice anesthetized by inhalation of 3% isoflurane/O_2_ and maintained at 2%. Then, the antibiotic Baytril (5 mg/kg; Bayer) and the anti-inflammatory/analgesic Meloxicam (300 µg/kg: VITA Laboratories) were subcutaneously administrated. After cleaning the abdominal region with ethanol 70% and saline 0.9%, a skin incision was made and the uterine horns were exposed. The lateral ventricle (LV) was visualized using cold light trans-illumination, and the *StarTrack* plasmid mixture was injected into the LV (0.5 µg/µL in distilled water) using Fast Green (0.2) to confirm the successful filling of the LV. To label pallial progenitors, the positive electrode was placed on the dorsal part of the injected LV to aim the negatively charged DNA. Five square electrical pulses of 35 mV for 50 ms were delivered at 950 ms intervals. Finally, the embryos were returned to the abdominal cavity and the incision was closed. Dams were placed in a sterile surgery area and maintained at 37 °C to reduce the risk of post-surgery infection. The embryos were allowed to develop until the selected age of analysis (P30). To avoid damaging the normal development of the brain (such as hydrocephalus, ectopias, heterotopias, or traumatic injury), which could modify neural cells’ behavior, proper microinjection was performed and a specific electroporation voltage was selected for the E14 embryonic stage [39]. To induce the *Cre*-recombinase activity and to remove the non-integrated plasmid copies, a single intraperitoneal injection of Tamoxifen (Sigma-Aldrich T5648-1 G, 20 mg/mL in corn oil) was administrated at P8 (7.5 mg/kg body weight).

### 2.4. Tissue Processing and Immunohistochemistry

At P30, mice were anesthetized with pentobarbital (Dolethal, 40–50 mg/kg) and transcardially perfused with 4% paraformaldehyde (PFA). Brains were removed and post-fixed for 1 h with 4% PFA in 0.1 M phosphate buffer (PB). Brains were vibratome sectioned at 50 µm in the coronal plane.

To identify the neural cell type of *StarTrack* labeled cells, we selected the following primary antibodies (Table 1): alpha-type platelet-derived growth factor receptor (PDGFRα) as the NG2-glia lineage marker; oligodendrocyte transcription factor 2 (Olig2) as the oligodendroglial lineage marker; adenomatous polyposis coli (APC) as the oligodendrocyte marker; S100 calcium-binding protein beta (S100β) as the astrocyte marker; and chicken ovalbumin upstream promoter transcription factor-interacting proteins 2 (CTIP-2) as the cortical V-VI layer marker. After washing and permeabilizing the tissue with phosphate-buffered saline containing Triton-X 100 (PBS-T) at 0.5% and PBS-T 0.1%, sections were incubated in a blocking buffer of 5% normal goat serum (NGS) in PBS-T 0.1% for at least 2 h. Primary antibodies were diluted in blocking buffer at selected concentrations (see Table 1) and incubated overnight at 4 °C. Sections were washed in PBS-T 0.1% 6 times and then incubated in secondary antibody for 2 h with Alexa-Fluor 633 goat anti-rabbit or 647 goat anti-mouse or anti-rat depending on the species of animal in which the primary antibody was raised (see Table 1). Sections were washed with PBS-T 0.1% and PBS 1x and mounted onto glass slides with Mowiol (Polysciences, Inc., Warrington, PA, USA).

### 2.5. Image Analysis and Processing

Sections were examined under epifluorescence microscopy using the appropriate filter cubes: GFP (FF01-473/10), mCherry (FF01-590/20), and Cy5 (FF01-628/40-25). Mosaic images were acquired using a TCS-SP5 confocal microscope (Leica, TCS-SP5). The six XFPs were taken in separated light wavelength channels, avoiding overlap between them. The confocal lines were between 15% and 30% intensity, and the wavelengths of excitation (Ex) and emission (Em) were (in nanometers): mT-Sapphire (Ex: 405; Em: 520–535), mCerulean (Ex: 458; Em:468–480), EGFP (Ex:488; Em: 498–510), YFP (Ex:514; Em: 525–535), mKO (Ex: 514; Em: 560–580), mCherry (Ex: 561; Em: 601–620), and Alexa Fluor 633/647 (Ex: 633; Em: 650–760). We used LasX software (Leica Application Suite X, Version 3.5.1, Wetzlar, Germany) to obtain the maximum projection of the images.

Clonal analysis was performed using a Fiji macro developed in the Scientific and Microscopy Image Unit of the Cajal Institute (Spain). After rostro-caudal sorting of the slice images, the macro generated a barcode for every cell depending on the presence or absence of the XFPs (YFP, mKO, mCerulean mCherry, mT-Sapphire, and EGFP). Later, through visual analyses, we determined the nuclear or cytoplasmic location of the XFPs. Cells with the same fluorophore code and the same fluorophore location were classified as sibling cells belonging to the same clone. To determine clones composed of different cell types, we used only those clones with XFP color combinations with less than 1% frequency. The cellular type was classified based on both their morphology and immunohistochemical markers. The electroporation site was delineated with the slices between the first and last sections containing fluorescent cells. The first slice with *StarTrack* labelled cells was considered as “0 µm”, increasing 50 µm (for the thickness of the sections) successively for every slice until the end of the labeling.

### 2.6. Statistical Analysis

Statistical comparisons were performed using both RStudio and Graphpad Prism (RStudio version 1.4.1106 and GraphPad Prism 6.0, San Diego, CA, USA). A non-Gaussian data distribution was obtained using the Kolmogorov–Smirnov test with Dallal Wilkinson–Liffie for the *p*-value. Then, the non-parametric Mann–Whitney test was used to compare two groups. To compare more than two groups, the non-parametric Kruskal–Wallis test was used. Data were represented using RStudio (version 1.4.1106). Considering a 95% confidence interval, statistical significance was determined at the critical values * *p*  <  0.05, ** *p*  <  0.01, and *** *p* <  0.001 reported in the text.

## 3. Results

### 3.1. Glial Cell Progeny Derived from Pallial RGC Progenitors in Lower Cortical Layers

**Figure 1:**
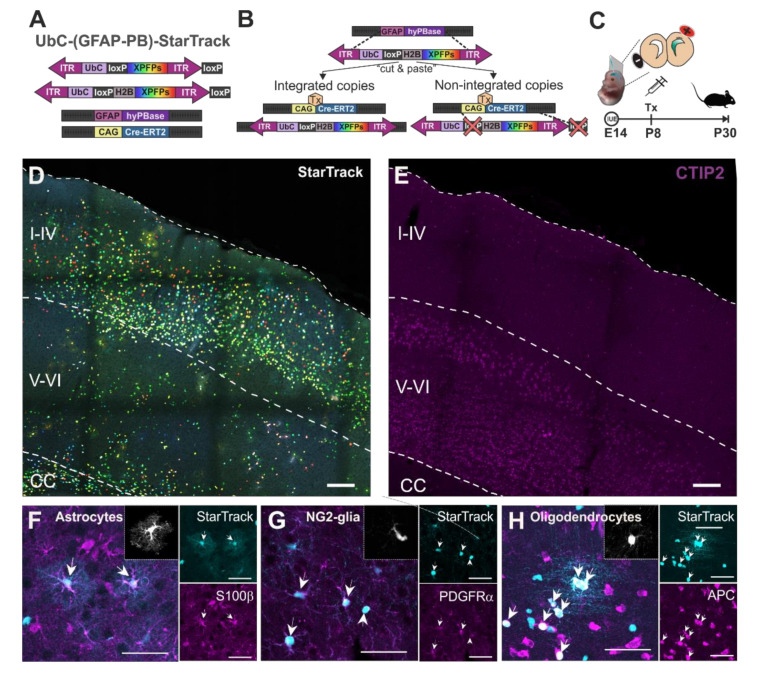
Adult glial cell progeny of targeted pallial E14-RGC progenitors with *UbC-(GFAP-PB)-StarTrack* in lower cortical layers. (**A**) Scheme of *UbC-(GFAP-PB)-StarTrack*, a genetic tool based on the *PiggyBac* system that enables the targeting of individual RGC progenitors to follow their complete cell progeny, independently of their lineage. This technique consists of the integration of 12 piggyBac plasmids under the *UbC-*promoter that encode up to 6 different fluorescent proteins (XFPs), located at the cytoplasm and/or the nucleus thanks to histone H2B. (**B**) The hyperactive *PiggyBac* transposase under the human *GFAP* promoter (*GFAP*-hyPBase) recognizes the inverted terminal repeats (ITR) of *PiggyBac* plasmids, allowing it to stochastically integrate copies specifically in RGC progenitors at a single cell level and follow their complete cell progeny, independently of their lineage. In addition, plasmids are floxed by two *lox-P*, allowing the removal of non-integrated copies thanks to the *Cre-loxP* system after tamoxifen (Tx) intraperitoneal injection. (**C**) Analysis of adult (P30) cell-derived progeny after targeting pallial RGC progenitors at E14 by in utero electroporation (IUE) with *UbC-(GFAP-PB)-StarTrack.* Tamoxifen was administered at P8 to remove the non-integrated plasmids. (**D**) Adult cortical cell progeny of pallial RGC progenitors after targeting with *StarTrack* at E14. (**E**) CTIP2+ cells to delineate the lower cortical layers (V-VI). (**F**) *StarTrack* labeled astrocytes expressing S100β. Inset shows the astrocyte morphology in a rendered image. (**G**) *StarTrack* labeled NG2-glia expressing PDGFRα+. Inset shows the NG2-glia morphology in a rendered image. (**H**) *StarTrack* labeled oligodendrocytes expressing APC+. Inset shows the oligodendrocyte morphology in a rendered image. Tx: tamoxifen; I-IV: upper cortical layers; V-VI: lower cortical layers; CC: corpus callosum. Scale bars in (**C**,**D**): 100 µm; in (**E**–**G**): 50 µm.

Single pallial RGC progenitors in the ventricular zone (VZ) were targeted by IUEs at E14 with *UbC-(GFAP-PB)-StarTrack* (Figure 1A–C). The *UbC-(GFAP-PB)-StarTrack* strategy [32] enables the targeting of individual RGC progenitors to follow their complete cell progeny, independently of their cell type. This technique is a modification of *UbC-StarTrack* [33], a multicolor genetic tool based on the integration of 12 piggyBac plasmids that encode up to 6 different fluorescent proteins (XFPs), located at the cytoplasm and/or the nucleus (Figure 1A). The expression of XFP plasmids is driven by the *UbiquitinC* promoter (*UbC* promoter), which drives consistent expression in all neural populations, allowing clonal relationships to be established between sibling cells [40]. The integration of XFP reporters in NPCs is carried out by the hyperactive *PiggyBac* transposase under the control of the human *GFAP* promoter (*GFAP*-hyPBase). In addition, plasmids are floxed by two *lox-P*, allowing the removal of non-integrated copies due to the *Cre-loxP* system (Figure 1B). The *StarTrack* mixture was injected into the LV at E14 and electroporated into RGC progenitor cells sited in the dorsal VZ (Figure 1C). To eliminate episomal copies [33], tamoxifen was intraperitoneally injected at P8, and brains were analyzed at P30 (Figure 1C). Cell type identification of *StarTrack* labeled cells (Figure 1D) was performed using both morphological features and immunohistochemical markers. As the *StarTrack* uses six different fluorescent proteins, the unique light wavelength channel available for immunohistochemistry was the far-red (Figure 1E–H). CTIP2 antibody was used to specifically delineate the layers V and VI (lower cortical layers; Figure 1E) since at P30 the cell progeny of E14-RGC progenitors were located throughout all the cortical layers (Figure 1D). Astrocytes were identified by their complex morphology with radial primary processes from the soma and their gradual branching into finer processes generating a dense network of terminal processes (Figure 1F). NG2-glia were recognized by their small cell bodies with diverse morphology and multibranched processes (Figure 1G). Finally, oligodendrocytes were recognized by their both round and small nuclei with aligned processes (Figure 1H). Even the *StarTrack* labelled cells were first identified by their morphological characteristics, and cell types were confirmed with the expression or the absence of immunohistochemical markers. Astrocytes were identified with S100β antibody (Figure 1F), NG2-glia were validated with the PDGFRα marker (Figure 1G), and oligodendrocytes were identified by the expression of APC antibody (Figure 1H). Thus, pallial E14-RGC progenitors generate different glial cell types in lower cortical layers, including astrocytes, NG2-glia, and oligodendrocytes.

### 3.2. Cell Fate Potential of Single Pallial E14-RGC Progenitors

The clonal relationships of labelled cells were determined after the identification of the color codes in 176,357 *StarTrack* labeled cells in both the cortex and corpus callosum (CC) from three animals. First, a six-digit barcode was generated for each cell, depending on the presence or absence of the XFPs (1, YFP; 2, mKO; 3, mCerulean; 4, mCherry; 5, mT-Sapphire; 6, EGFP; and 0, absence of fluorescent signal). Then, through a supervised visual analysis, we determined the cytoplasmic/nuclear cell location of the XFPs to define the final 12-digit barcode for each sibling cell (i.e., 123456 123456). Thus, the first six numbers were related to the cytoplasmic XFP labeling, while the next six were related to the nuclear XFP labeling. Therefore, cells with the same color code and the same location of fluorophores were classified as sibling cells belonging to the same clone. Furthermore, since the transposase can integrate a variable number of plasmid copies, the intensity of fluorophores in each cell was an additional parameter to accurately define a clone. Clones composed of sibling cells belonging to different glial cell types were selected according to the less frequent color code combinations. We studied 46 different clones (Table S1), composed of a total of 783 glial cells, located in the lower cortical layers, although some of their sibling cells occupied upper cortical layers or CC that were included in the analyses. Additionally, the rostro-caudal (R-C) cell dispersion was established as the total distance between the first and the last cells belonging to a clone, considering that the thickness of the sections was 50 µm. Later, cell type was determined by the morphology and cell type immunohistochemistry markers. We detected clones comprising 4 to 49 cells, extended from 50 µm up to 350 µm.

**Figure 2:**
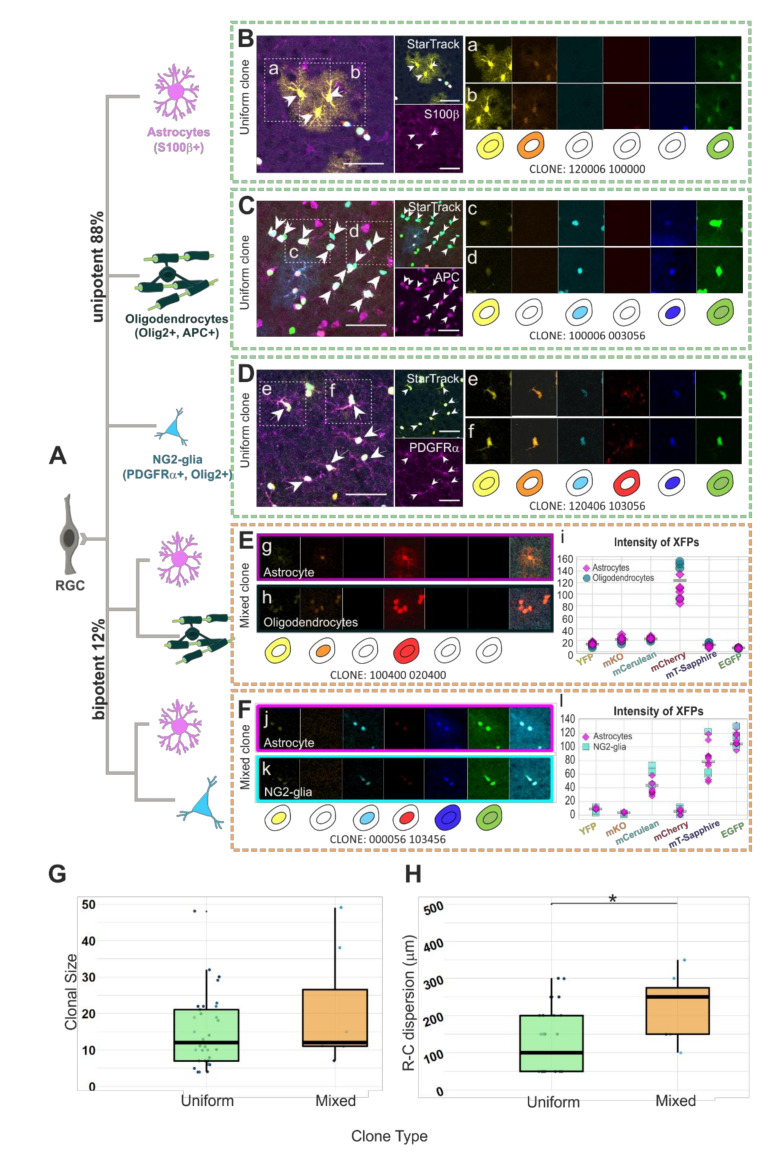
Clonal analysis of glial cell progeny derived from single pallial E14-RGC progenitors in lower cortical layers. (**A**) After targeting single RGC progenitors, we observed uniform clones (88%, *n* = 40 clones) composed of cells belonging to one glial lineage and mixed clones (12%, *n* = 6 clones) composed of two glial cell types. (**B**) Sibling *StarTrack* labeled astrocytes S100β+. (**a**,**b**) Numeric and graphical representation of the color code of an astroglial clone according to the cytoplasmic/nuclear (123456 123456) presence of 1, YFP; 2, mKO; 3, mCerulean; 4, mCherry; 5, mT-Sapphire; and 6, EGFP; or 0, their absence. (**C**) Sibling *StarTrack* labeled oligodendrocytes APC+. (**c**,**d**) Numeric and graphical representation of the color code of an oligodendrocyte clone. (**D**) Sibling *StarTrack* labeled NG2-glia PDGFRα+. (**e**,**f**) Numeric and graphical representation of the color code of a NG2-glia clone. (**E**) Mixed clone of oligodendrocytes and astrocytes originating from the same progenitor. (g-h) Numeric and graphical representation of the color code of a mixed astroglial+oligodendroglial clone. (**i**) Color intensity of the 6 XFPs for every sibling cell of the clone. (**F**) Mixed clone composed of NG2-glia and astrocytes. (**j**,**k**) Numeric and graphical representation of the color code of a mixed astroglial+NG2-glia clone. (**l**) Color intensity of the 6 XFPs for every sibling cell of the clone. (**G**) No significant clonal size differences between mixed clones (22.67 ± 6.77c/clone) and uniform clones (14.55 ± 1.54 c/clone). (**H**) Larger rostro-caudal (R-C) cell dispersion between cells of mixed clones (233.3 ± 38.01 µm) than between uniform clones (130.06 ± 75.85 µm). *n* = 46 clones from 3 animals. * *p* < 0.05; Scale bar: 50 µm.

Most single pallial E14-RGC progenitors gave rise to uniform clones of sibling cells (Figure 2A, 88%; *n* = 40) of the same cell type, either astrocytes (Figure 2B(a,b)), oligodendrocytes (Figure 2C(c,d)), or NG2-glia (Figure 2D(e,f)). We also classified as uniform clones those formed by NG2-glia and oligodendrocytes, since some NG2-glia can act as oligodendrocyte precursor cells (OPCs). The rest of the clones (12%; *n* = 6) were formed by sibling cells from two different glial cell types (mixed clones). Half of the mixed glial clones were composed of both astrocytes (Figure 2E(g)) and oligodendrocytes (Figure 2E(h)). Figure 2E shows a mixed clone formed by astrocytes and oligodendrocytes with the color code 100400 020400, meaning the presence of YFP in the cytoplasm, mKO in the nucleus, and mCherry in both the cytoplasm and nucleus. Furthermore, since the XFP intensity values reinforce their accurate clonal identity, the XFP intensity for sibling cells differed by less than 80 points (Figure 2E(i)). The rest of the mixed glial clones (Figure 2F) were composed of both astrocytes (Figure 2F(j)) and NG2-glia (Figure 2F(k)). The mixed astrocyte/NG2-glia clone in Figure 2F shows YFP, mCerulean, and mCherry in the nucleus, while in both the cytoplasm and nucleus, mT-Sapphire and EGFP are displayed (color code: 000056 103456). The fluorophore intensity variation was less than 80 points within analyzed sibling cells, independently of their lineage (Figure 2F(l)). No significant clonal size differences were found between both types of clones (Figure 2G). Uniform clones were formed by 4 to 32 sibling cells per clone (14.55 ± 1.54 c/clone) and mixed clones were constituted of 11 to 49 sibling cells per clone (22.67 ± 6.77 c/clone). Although mixed and uniform clones were of a similar number of cells, the R-C cell dispersion of those cells (Figure 2H) was higher (*p* = 0.0114) in mixed clones (233.3 ± 93.09 µm) than in uniform clones (130.06 ± 75.85 µm).

Among the glial progenitors, most of the pallial E14-RGC progenitors generate uniform glial clones, and few of them produce mixed clones. This suggests the existence of pallial E14-RGC progenitors restricted to one glial lineage, while few of them maintain the potential to generate different glial cell types.

### 3.3. Clonal Size and Rostro-Caudal Dispersion of the Derived Cell Progeny from Single Pallial E14-RGC Progenitors

**Figure 3:**
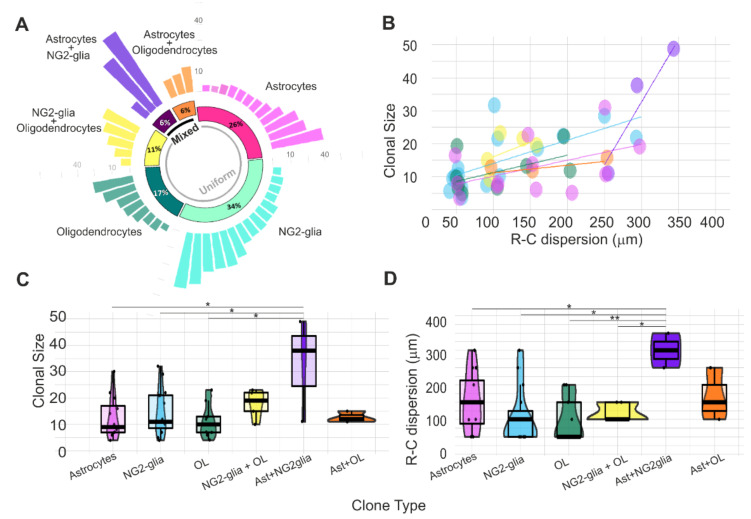
Clonal size and R-C cell dispersion of the glial-derived cell progeny from single pallial E14-RGC progenitors. (**A**) Clonal analysis of mixed and uniform clones from single pallial E14-RGC progenitors: 26% clones of astrocytes (*n* = 12 clones), 34% NG2-glia (*n* = 15 clones), 17% oligodendroglial clones (*n* = 8 clones), and 11% NG2+oligodendroglial clones (*n* = 5 clones). Half of the mixed clones comprised NG2 and astrocyte sibling cells, and the other half comprised oligodendrocyte and astrocyte sibling cells. (**B**) Increment in the clonal size related to the R-C cell dispersion. Purple: astrocyte clones; blue: NG2-glia clones; yellow: NG2-glia+oligodendrocyte clones; green: oligodendrocyte clones. (**C**) Number of sibling cells per clone type. (**D**) R-C cell dispersion of clones sorted by the type of clone. R-C dispersion in B and D corresponds to the extension of sibling cells in microns. Every point represents a clone. Ast: astrocytes; OL: oligodendrocytes. * *p* < 0.05; ** *p* < 0.01.

Retrospective *StarTrack* analysis revealed that the glial progeny derived from single pallial E14-RGC progenitors (Figure 3A) comprised uniform clones composed exclusively of astrocytes (26%), NG2-glia (34%), oligodendrocytes (17%), or NG2-glia+oligodendrocytes (11%) and mixed clones constituted by astrocytes+NG2-glia (6%) or astrocytes+oligodendrocytes (6%). The R-C cell dispersion increased with the number of cells per clone (clonal size, Figure 3B).

There was no significant difference in the average clonal size between the different uniform clones (Figure 3C): astrocytes (11.58 ± 8.251 cell/clone), NG2-glia (16.58 ± 8.607 cell/clone), oligodendrocytes (11.88 ± 6.424 cell/clone), and oligodendrocytes+NG2-glia (14.40 ± 6.504 cell/clone). However, we found significant differences in the size of astroglial+NG2-glia mixed clones in relation to the following uniform clones: astrocytes (*p* = 0.048), NG2-glia (*p* = 0.041), and oligodendrocytes (*p* = 0.019; Figure 3C). While NG2-glia or astroglial clones numbered up to 30 sibling cells, oligodendrocytes or oligodendrocytes+NG2-glia clones reached up to 20 sibling cells (Figure 3C). Mixed clones formed by astrocytes+NG2-glia were bigger (32 ± 19.55 cells per clone) than astrocyte+oligodendrocyte clones (12.67 ± 2.082 cells per clone) (Figure 3C).

The average R-C cell dispersions of uniform clones did not show statistical differences between them (Figure 3D): 170 µm (170 ± 78.88 µm) in astroglial clones; 120 µm (120.8 ± 86.49 µm) in NG2-glia clones, 100 µm in oligodendrocyte clones (100 ± 66.14 µm), and 110 µm (125 ± 28.87 µm) in oligodendrocyte+NG2-glia clones. Nonetheless, significant differences were found between astroglial+NG2glia mixed clones and the following uniform clones: astrocytes (*p* = 0.049), NG2-glia (*p* = 0.011), oligodendrocytes (*p* = 0.0045), and oligodendrocytes+NG2-glia (*p* = 0.0286; Figure 3D). Cells of astroglial clones and NG2-glia clones spread along the R-C axis from 50 to 300 µm, while the maximum cell dispersion of oligodendrocyte clones or oligodendrocyte+NG2-glia clones reached up to 150–200 µm (Figure 3D). Then, while astroglial clones were smaller than others, their sibling cells were more dispersed in the R-C axis. In contrast, NG2-glia clones were bigger but with less R-C cell dispersion.

### 3.4. Cortical Dispersion of the Glial Cell Progeny derived from Single Pallial E14-RGC Progenitors

**Figure 4:**
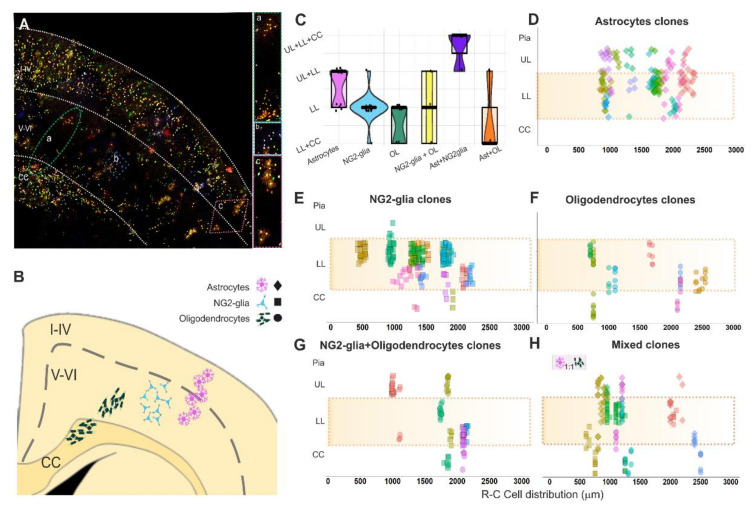
Clonal cell dispersion of glial cell progeny of single pallial E14-RGC progenitors. (**A**) Cortical cell progeny of single pallial E14-RGC progenitors labeled with *StarTrack* in a coronal section. (**a**) Green oval line: magnification of the oligodendroglial clone located in both the lower layers and CC. (**b**) Blue square line: magnification of an NG-glia clone exclusively located in lower cortical layers. (**c**) Pink diamond line: magnification of an astroglial clone located in upper and lower cortical layers. (**B**) Scheme showing the clonal arrangement of sibling cells. (**C**) Cortical dispersion of glial progeny from single pallial NPCs. Each point represents a clone. (**D**) Clonal dispersion of sibling astrocytes. (**E**) Clonal dispersion of sibling NG2-glia. (**F**) Clonal dispersion of sibling oligodendrocytes. (**G**) Clonal dispersion of sibling NG2-glia+oligodendrocytes. (**H**) Clonal dispersion of mixed clones. In D–H, every point represents a cell belonging to a clone that is represented by one different color. The shadow orange areas define the lower cortical layers. R-C cell distribution: location in the rostro-caudal axis of cells that compose a clone, considering “0” as the first most rostral slice with *StarTrack* labeled cells and increasing by 50 µm (thickness of the sections) successively for every slice until the end of the labeling. Ast: astrocytes; OL: oligodendrocytes; CC: corpus callosum; LL.: lower cortical layers (V-VI); UL.: upper cortical layers (I-IV).

Glial clones were analyzed in the lower cortical layers, although it was frequent to find sibling cells in the upper cortical layers and CC, which were also included in the clonal analyses (Figure 4A,B). Most clones were restricted to one cell type, although their sibling cells could be widespread throughout the different cortical layers outside of layers V and VI. Sibling astrocytes were either sparsely distributed or forming vertical arrangements (Figure 4A(c),B) in the lower layers or in both the lower and upper layers (Figure 4C,D). NG2-glia clones formed cell clusters restricted to lower cortical layers (Figure 4A(b),B–E), although in some clones, sibling cells were also distributed in the upper layers or CC (Figure 4C,E). Cells in oligodendrocyte clones were arranged in lower cortical layers or even expanded into the CC (Figure 4A(a),C,F). Sibling cells in most of the oligodendrocyte+NG2-glia clones were spread into the upper layers or CC (Figure 4C,G). All mixed clones crossed the limits of the lower cortical layers (Figure 4H). However, most of the astrocyte+NG2glia mixed clones were sited in the upper layers, lower layers, and CC, while astroglial+oligodendroglial mixed clones spread to the CC (Figure 4C,H). We noticed that in mixed clones, astrocytes were located in layers above their sibling oligodendrocytes or NG2-glia (Figure 4H). Further, mixed clones were composed of the same number of astrocytes and oligodendrocytes (Figure 4H), but not those formed of astrocytes and NG2-glia.

To sum up, our data revealed the presence of two different types of uniform clones, one regionally restricted to lower cortical layers and other that spread out that limit. However, mixed clones were dispersed across different cortical layers.

## 4. Discussion

In the present study, we tracked single pallial GFAP-expressing progenitors (RGC-E14 progenitors) to decipher the clonal relationships of their adult glial progeny in lower cortical layers. Our data revealed the presence of both uniform clones composed of just one cell type and mixed clones with two glial cell types. Uniform clones comprised either astrocytes, oligodendrocytes, or NG2-glia. Further, since some NG2-glia can give rise to OPCs and those, in turn, to oligodendrocytes, we decided to classify as uniform those clones formed by NG2-glia and oligodendrocytes. Mixed clones contained sibling cells from two glial cell types: astrocytes+oligodendrocytes or astrocytes+NG2-glia. Most clones were uniform, although the number of cells per clone was similar between the uniform and mixed clones. However, the R-C cell dispersion of mixed clones was larger than that of uniform clones. Even when clones were selected in the lower cortical layers, some of their sibling cells were located in other cortical regions. Most NG2-glia clones were restricted to the lower layers, while some astroglial and oligodendroglial clones spread into the upper layers and CC, respectively.

Currently, there is far less knowledge of gliogenesis compared to neurogenesis [2,4,8,12,29,35,41]. Neuronal fate acquisition occurs at specific timings according to intrinsic and extrinsic factors [6,15,17]. Considering the temporal waves of cortical neurogenesis, we wondered whether this pattern occurs in gliogenesis. Moreover, although it is known how cortical progenitors give rise to neurons and some macroglial cell types, the clonal relationships between glial cell types remain partially unknown. Some studies have focused on clonal aspects of astrocyte generation [30,38,42,43,44,45,46,47,48] and few of them have focused on oligodendrocytes [38,48,49] or NG2-glia [47]. Recent single-cell analysis revealed that glial cells in different layers exhibit distinct transcriptional profiles [36,37]. Moreover, neuronal layers play an essential role in the establishment or maintenance of layer-specific properties of cortical astroglia [36,38]. Furthermore, neocortical embryonic RGCs produce oligodendrocyte clones more frequently located in the lower cortical layers and CC [38]. In addition, NG2 cells sited in upper cortical layers are highly ordered [40]. Thus, due to the aforementioned differences, we focused on those glial clones located in lower cortical layers.

We reported uniform clones composed of either astrocytes, oligodendrocytes, or NG2-glia. These results match with previous studies using *StarTrack* and Mosaic Analysis with Double Markers (MADM) from embryonic progenitors that showed clones composed exclusively of astrocytes [30,45], oligodendrocytes [38], or NG2-glia [31]. However, other studies with MADM did not report any cortical clones containing exclusively oligodendrocytes [45], because RGCs generate diverse cell fate ratios during different embryonic temporal windows. Furthermore, while some researchers showed that NG2-glia derived from two waves of ventral embryonic progenitors and postnatal dorsal progenitors [11], we and others [31,50] noticed that those cells also derived from pallial E14 progenitors. Recently, Li et al. [51], combining single-cell RNA-Seq with intersectional lineage analyses, reported that most oligodendrocytes derived from multipotent IPCs around E16.5 [51].

The glial clonal size varied from 4 to 49 cells per clone [38,45]. Astroglial clones from the lower cortical layers were composed of 4–30 cells. This differs from the 50 cells per clone reported in all cortical layers in our previous *StarTrack* analyses [30], due to the clonal size in upper layers being larger than that in lower layers [43]. Consistently with former works, NG2-glia clones were composed of 4–32 cells at P30 [31]. However, at P270, NG2-glia form the largest clonal clusters of the brain (up to 340 cells per clone) [31]. This increment of the clonal size in adulthood could indicate that NG2-glia remain quiescent in the adult mouse brain, increasing their proliferative rate or decreasing apoptosis when needed [52]. These differences in clonal size could be explained by the different proliferative rates of intermediate precursors. MADM analysis showed that RGPs produce a stochastic number of intermediate astrocyte or oligodendrocyte precursors that can differentiate into two or three sibling astrocytes or six sibling oligodendrocytes, respectively [38]. Those amplifying intermediate progenitors have also been observed in D*rosophila* brains [53], where gliogenic precursors produce around nine glial cells per clone [54,55]. Furthermore, clonal lineage analysis of PSC-derived brain organoids revealed that macaque progenitor cells cease their progenitor expansion phase earlier in development than humans [56].

Although glial clones were selected from lower cortical layers, sibling astrocytes spread into vertical arrangements along the upper and lower cortical layers. This could be explained by intermediate astrocyte precursor cells that differentiate along cortical layers [38]. This cellular disposition could have functional implications, as occurs with the functional columnar arrangement of neurons in the adult neocortex [4]. In fact, the molecular pattern and morphology of cortical astrocytes display differences that could have consequences in their structural interaction with synapses (enrichment in upper cortical layers) and might modulate glutamate clearance and synaptic plasticity [36]. Moreover, oligodendrocytes belonging to the same clone, located in both the lower layers and CC, were arranged like cell patches following the process of RGCs. Those subclusters were observed using MADM, where RGPs produced intermediate OPCs that differentiate into oligodendrocytes [38]. In addition, the arrangement of sibling oligodendrocytes could be related to the axonal myelination of neurons belonging to the same cortical radial unit [4]. In contrast to astrocytes and oligodendrocytes, sibling cells of NG2-glia clones in lower cortical layers were regionally restricted. Although NG2-glia are distributed throughout white and grey matter, they mainly differentiate into oligodendrocytes in the CC [57]. NG2-glia in the grey matter could serve as a reservoir to reactivate their proliferation at any time to replace the loss of oligodendrocytes [58]. This cohesive cluster distribution of NG2-glia clones was also reported in daughter cells from zebrafish progenitors, suggesting a rapid cell proliferation and limitation of daughter cell dispersion [59].

Previous *StarTrack* analyses deepened insights into the clonal relationships of astrocytes and NG2-glia [30,31]. Nonetheless, the complete cell progeny of progenitors was not visible when the *GFAP* promoter was switched off [31]. In this study, we exclusively tagged single pallial GFAP-expressing progenitors with *UbC-(GFAP-PB)-StarTrack* to track their complete cell progeny independently of their lineage, even when *GFAP* promoter was switched off. We described mixed clones composed of two different glial cell types. Uniform and mixed glial clones confirmed the existence of monopotent and bipotent pallial RGC progenitors in the VZ at E14 [1,16,60,61,62,63]. Histological analyses, cell transplantations, and in vitro studies have claimed progressive competence restriction of progenitors, being multipotent at earlier stages and reducing their cell potential with development [21,22]. However, genetic fate-mapping studies [34] argued that fate-restricted progenitors are present early in development, as we stated in this study [15,17].

Lately, the molecular profile of NPCS suggests that they are a heterogeneous pool [61,62,63,64], although it is still unclear whether their cell potential is defined by intrinsic or extrinsic factors. Several lines propose that most neurons, oligodendrocytes, and astrocytes are not the direct progeny of RGCs, but instead originate from intermediate progenitor cells [51]. Single-cell RNAseq revealed that astrogliogenesis is a dynamic process throughout a transitional progenitor population, while oligodendrogenesis is fate-restricted with a primitive-OPC intermediate population before OPC [65,66]. Besides this, scRNA-seq on EGFR+ cells isolated from the human frontal cerebral cortex, at the onset of gliogenesis, revealed the coexistence of 12 subtypes of progenitors with molecular features of either neuronal, oligodendroglial, or astroglial progenitors [16]. In addition, the molecular profile of single progenitors revealed some clusters of neural progenitors with either quiescent or proliferative properties [16,61,62,63,65]. Altogether, our results support the heterogeneity of pallial progenitor cells in their cell fate, proliferative potential, and location.

## 5. Conclusions

This work contributes to deciphering the heterogeneity of neural progenitor cells. We provide new clues to unravel the diversity of pallial single E14-RGC progenitors that exhibit uni- and bi-cell potential. Further studies combining clonal analysis with the molecular profile of each clone could help to better our knowledge of the potential of neural progenitor cells and neural cell diversity.

## Figures and Tables

**Table 1:** List of primary and secondary antibodies for the molecular characterization of neural cells.

Antibody	Abbr.	Use	Species	Source	Reference
Adenomatous polyposis coli	APC	1:300	Mouse	Calbiochem	OP80
Chicken ovalbumin upstream promoter transcription factor-interacting proteins 2	CTIP2	1:500	Rat	Abcam	Ab18465
Oligodendrocyte transcription factor 2	Olig2	1:500	Rabbit	Millipore	AB9610
Alpha-type platelet-derived growth factor receptor	PDGFRα	1:300	Rabbit	Cell Signalling	31745
S100 calcium binding protein beta	S100B	1:500	Rabbit	Abcam	Ab41548
Secondary Ab Alexa fluor 647	anti-mouse	1:1000	Goat	ThermoFisher	A21236
Secondary Ab Alexa fluor 633	anti-rabbit	1:1000	Goat	ThermoFisher	A21070
Secondary Ab Alexa fluor 647	anti-rat	1:1000	Goat	Invitrogen	A21247

## Supplementary Materials

The following are available online at https://www.mdpi.com/article/10.3390/cells10113237/s1, Table S1: Clones derived from E14-GFAP-progenitor cells.
