# Supplementary material for: Gliogenic Potential of Single Pallial Radial Glial Cells in Lower Cortical Layers
Source: Cells. 2021 Nov 19;10(11):3237. doi: 10.3390/cells10113237 (PMC8621618; doi:10.3390/cells10113237)
Supplement: Supplementary file 1 [file cells-10-03237-s001.zip › cells-1366049-supplementary.pdf]

Supplementary material.

Gliogenic Potential Of Single Pallial Radial Glial Cells in Lower Cortical Layers

Ana Cristina Ojalvo-Sanz 1,# and Laura López-Mascaraque 1,*

Table S1. Clones analyzed in the progeny of E14-GFAP-progenitor cells

Animal	Clone_digital code *	Clone cell type	Clone type	Clonal size	Number cells and location **	R-C dispersión ***	Cortical dispersion ****	IHC *****
A1	10a_103056 103056	Astrocytes	uniform clone	22	4- UL; 18-LL	150	LL+UL	Olig2
A1	1c_120000 003056	Astrocytes	uniform clone	7	6-LL	100	LL	Olig2
A1	2a_003006 003056	Astrocytes	uniform clone	10	10-LL	250	LL	Olig2; NeuN
A1	4a_000400 120400	Astrocytes	uniform clone	20	7-UL; 13-LL	300	LL+UL	Olig2; NeuN; APC
A1	9a_123056 123456	Astrocytes	uniform clone	30	30- LL	250	LL	NeuN; S100β; PDGFRα
A2	4a_120000 123400	Astrocytes	uniform clone	7	7-LL	150	LL	APC
A2	5b_123000 123006	Astrocytes	uniform clone	4	4-LL	50	LL	S100β
A2	6a_003000 000406	Astrocytes	uniform clone	14	10-UL; 4 LL	150	LL+UL	Olig2; APC
A3	10a_123000 12000	Astrocytes	uniform clone	6	5-UL; 1-LL	200	LL+UL	Olig2; S100β
A3	12c_000056 120056	Astrocytes	uniform clone	8	8_LL	100	LL	S100β
A2	2b_000400 003456	Astrocytes	uniform clone	6	2-UL; 4-LL	100	LL+UL	PDGFRα
A2	8a_100000 000456	Astrocytes	uniform clone	12	7-UL; 5-LL		LL+UL	APC; Olig2
A1	4b_120000 000400	NG2-glia	uniform clone	18	8-LL; 3-CC	150	LL + CC	Olig2; PDGFFRa; APC
A1	6a_003400 000400	NG2-glia	uniform clone	11	11-LL	50	LL	Olig2
A1	7a_020006 000406	NG2-glia	uniform clone	29	29-LL	250	LL	Olig2-APC-S100β
A1	9b_123000 100456	NG2-glia	uniform clone	5	5-LL	50	LL	PDGFRα
A2	10a_000000 123000	NG2-glia	uniform clone	14	14-LL	50	LL	PDGFRα
A2	1a_103000 023456	NG2-glia	uniform clone	21	22-LL	100	LL	S100β

Animal	Clone_digital code *	Clone cell type	Clone type	Clonal size	Number cells and location **	R-C dispersión ***	Cortical dispersion ****	IHC *****
A2	5a_120006 123006	NG2-glia	uniform clone	32	30-LL	100	LL+UL	PDGFRα, Olig2
A2	8b_100000 100456	NG2-glia	uniform clone	11	11-LL	100	LL	Olig2
A2	9a_023000 000400	NG2-glia	uniform clone	5	5-LL	50	LL	S100β
A3	15a_000400 000456	NG2-glia	uniform clone	21	24-LL	300	LL	Olig2; S100β
A3	15b_000456 000450	NG2-glia	uniform clone	12	6-LL; 6-CC	50	LL + CC	Olig2, PDGFRα
A3	4b_003406 000050	NG2-glia	uniform clone	11	12-LL	50	LL	Olig2
A3	4c_000400 003456	NG2-glia	uniform clone	22	22-LL	200	LL	Olig2, APC; PDGFRα
A3	5a_100000 000400	NG2-glia	uniform clone	7	7_LL	150	LL	PDGFRα
A3	6c_123400 100056	NG2-glia	uniform clone	7	2-LL; 4-CC	100	LL	Olig2
A1	1a_000000 123056	Oligodendrocytes	uniform clone	12	11-LL	200	LL	APC; Olig2; PDGFRα; S100β
A2	1b_100006 123456	Oligodendrocytes	uniform clone	19	10-LL; 8-CC	50	LL + CC	S100β
A2	2a_003000 000456	Oligodendrocytes	uniform clone	7	7-LL	50	LL	PDGFRα
A2	9a_023000 000400	Oligodendrocytes	uniform clone	5	5-LL	50	LL	PDGFRα
A3	12d_100000 120056	Oligodendrocytes	uniform clone	13	3-LL; 10-CC	150	LL + CC	S100β
A3	14a_120000 120006	Oligodendrocytes	uniform clone	23	6-LL; 17-CC	200	LL + CC	S100β, PDGFRα
A3	16a_020400 003456	Oligodendrocytes	uniform clone	7	7-LL	100	LL	Olig2, PDGFRα
A3	6a_100006 123456	Oligodendrocytes	uniform clone	4	4-LL	50	LL	S100β
A1	1b_120006 103050	NG2-glia +Oligodendrcoytes	uniform clone	19	NG2glia: 7-CC + OLs: 11-LL	150	LL+UL	APC
A3	3a_100006 123456	NG2-glia +Oligodendrcoytes	uniform clone	22	NG2glia: 19-UL + OLs: 3-LL	150	LL	S100β; Olig2

Animal	Clone_digital code *	Clone cell type	Clone type	Clonal size	Number cells and location **	R-C dispersión ***	Cortical dispersion ****	IHC *****
A2	8b_100000 100456	NG2-glia +Oligodendrcoytes	uniform clone	10	NG2glia: 6_LL + OLs: 4- CC	100	LL + CC	PDGFRα;
A2	12a_ 100400 120456	NG2-glia +Oligodendrcoytes	uniform clone	23	NG2glia:18-LL+ OL:5-CC	100	LL+CC	S100β; Olig2
A3	13a_000006 000400	NG2-glia +Oligodendrcoytes	uniform clone	15	NG2glia: 5-LL + OLs: 10-UL	100	LL	S100β; APC
A1	5a_000056 103456	NG2glia + Astrocytes	mixed clone	11	Ast: 9-LL; 3-UL + NG2glia: 5-LL	250	LL+UL	S100β; APC
A2	3a_120000 100000	NG2glia + Astrocytes	mixed clone	49	Ast: 8-UL; 21-LL;3-CC + NG2glia: 13-LL; 7 CC	350	LL+UL	S100β, APC, PDGFRα, Olig2
A2	7a_000006 003056	NG2glia + Astrocytes	mixed clone	38	Ast: 3-UL + NG2glia: 30-LL; 5-CC	300	LL+UL	S100β; Olig2
A1	3a_000000 103006	OLs + Astrocytes	mixed clone	15	Ast.: 2-UL; 6-LL + OL: 7-CC	250	LL + CC	APC; S100β
A3	1a_120406 123406	OLs + Astrocytes	mixed clone	11	Ast: 6-LL + OL: 5-CC	150	LL + CC	S100β; Olig2; APC
A3	8a_100400 020400	OLs + Astrocytes	mixed clone	12	Ast: 6-UL + OL: 6-LL	100	LL + CC	Olig2; S100β

* Twelve number code of the presence of YFP-1, mKO-2, mCerulean-3, mCherry-4, mT-sapphire-5, EGFP-6 or absence-0, first cytoplasm and later nuclear.

** Number of cells located in upper cortical layers (UL), lower cortical layers (LL) or corpus callosum (CC).Ast: astrocytes; OL: oligodendrocytes.

*** Total distance between the first and the last cells that compose a clone, considering that thickness of the sections are 50µm.

**** Dispersion of sibling cells that compose a clone in cortical layers.

***** Immunohistochemistry in section where sibling cells are located that help to corroborate or discard the cell type identified with morphological characteristics.
